# Adherence to Telemonitoring Therapy for Medicaid Patients With Hypertension: Case Study

**DOI:** 10.2196/29018

**Published:** 2021-09-06

**Authors:** Sulki Park, Hye-Chung Kum, Michael A Morrisey, Qi Zheng, Mark A Lawley

**Affiliations:** 1 Population Informatics Lab Texas A&M University College Station, TX United States; 2 Department of Industrial and Systems Engineering Texas A&M University College Station, TX United States; 3 Department of Health Policy and Management Texas A&M University College Station, TX United States; 4 Department of Epidemiology and Biostatistics Texas A&M University College Station, TX United States

**Keywords:** telemedicine, hypertension, Medicaid, blood pressure, pulse, telemonitoring, mobile phone

## Abstract

**Background:**

Almost 50% of the adults in the United States have hypertension. Although clinical trials indicate that home blood pressure monitoring can be effective in managing hypertension, the reported results might not materialize in practice because of patient adherence problems.

**Objective:**

The aims of this study are to characterize the adherence of Medicaid patients with hypertension to daily telemonitoring, identify the impacts of adherence reminder calls, and investigate associations with blood pressure control.

**Methods:**

This study targeted Medicaid patients with hypertension from the state of Texas. A total of 180 days of blood pressure and pulse data in 2016-2018 from a telemonitoring company were analyzed for mean transmission rate and mean blood pressure change. The first 30 days of data were excluded because of startup effects. The protocols required the patients to transmit readings by a specified time daily. Patients not transmitting their readings received an adherence reminder call to troubleshoot problems and encourage transmission. The patients were classified into adherent and nonadherent cohorts; adherent patients were those who transmitted data on at least 80% of the days.

**Results:**

The mean patient age was 73.2 (SD 11.7) years. Of the 823 patients, 536 (65.1%) were women, and 660 (80.2%) were urban residents. The adherent cohort (475/823, 57.7%) had mean transmission rates of 74.9% before the adherence reminder call and 91.3% after the call, whereas the nonadherent cohort (348/823, 42.3%) had mean transmission rates of 39% and 58% before and after the call, respectively. From month 1 to month 5, the transmission rates dropped by 1.9% and 10.2% for the adherent and nonadherent cohorts, respectively. The systolic and diastolic blood pressure values improved by an average of 2.2 and 0.7 mm Hg (*P*<.001 and *P*=.004), respectively, for the adherent cohort during the study period, whereas only the systolic blood pressure value improved by an average of 1.6 mm Hg (*P*=.02) for the nonadherent cohort.

**Conclusions:**

Although we found that patients can achieve high levels of adherence, many experience adherence problems. Although adherence reminder calls help, they may not be sufficient. Telemonitoring lowered blood pressure, as has been observed in clinical trials. Furthermore, blood pressure control was positively associated with adherence.

## Introduction

### Background

Hypertension affects nearly half of the adults in the United States, costs approximately US $131 billion annually, and is a major risk factor for cardiovascular disease and stroke [1-3]. Researchers have estimated the hazard ratios of cardiovascular events, stroke, and all-cause mortality to be 1.11-1.42, 1.28-1.40, and 1.02-1.13, respectively, for every 10 mm Hg increase in the ambulatory systolic blood pressure value [4]. Nonetheless, approximately only 1 in 4 adults with hypertension have their blood pressure under control [1].

Home blood pressure monitoring is an emerging strategy to help control hypertension, with many medical organizations recommending its use in diagnosis, distinguishing between blood pressure phenotypes, and ongoing hypertension management [5]. Several meta-analyses of published clinical trials have found evidence that home blood pressure monitoring can lead to clinically significant reductions in blood pressure values when accompanied by additional support services such as medication titration, education, and lifestyle counseling [6-9].

Although clinical trials are the gold standard, the reported results may not materialize in practice. Physicians have concerns about instrumentation quality, patient skills in taking readings, regular recording and transmission of results, and adherence to a regimen of routine measurement [7]. The 2010 and 2014 surveys of Canadian patients at a hypertension clinic where patients were encouraged to conduct home blood pressure monitoring found that only 39.2% and 40.6%, respectively, reported blood pressure more than 80% of the time [10]. Thus, poor patient adherence to daily monitoring and reporting could significantly undermine the positive effects observed in clinical trials.

### Objectives

The aims of this study are to (1) investigate how well Texas Medicaid patients adhere to daily blood pressure and pulse rate monitoring when supported by a daily telemonitoring services company, (2) determine whether an adherence reminder call intervention improves the daily transmission rate, and (3) investigate any association between daily adherence and blood pressure control.

We note that insurance coverage is a requisite for daily telemonitoring. Medicare began paying for home monitoring in November 2018 [11], and Texas Medicaid reimburses physician-prescribed home telemonitoring for hypertension for 60 days, with reauthorization for additional monitoring at physician request [12,13]. More generally, reimbursement rates for home monitoring services vary significantly among states and insurers and have an uncertain future [5]. Although the temporary support for telehealth services by the Centers for Medicare & Medicaid Services and private insurers as a result of the coronavirus pandemic may lead to permanent changes in the delivery of routine care [14,15], the future of home telemonitoring coverage is unclear. In this uncertain environment, the analysis of real-world telemonitoring implementations is of great importance.

## Methods

### Monitoring Protocol

**Figure 1:**
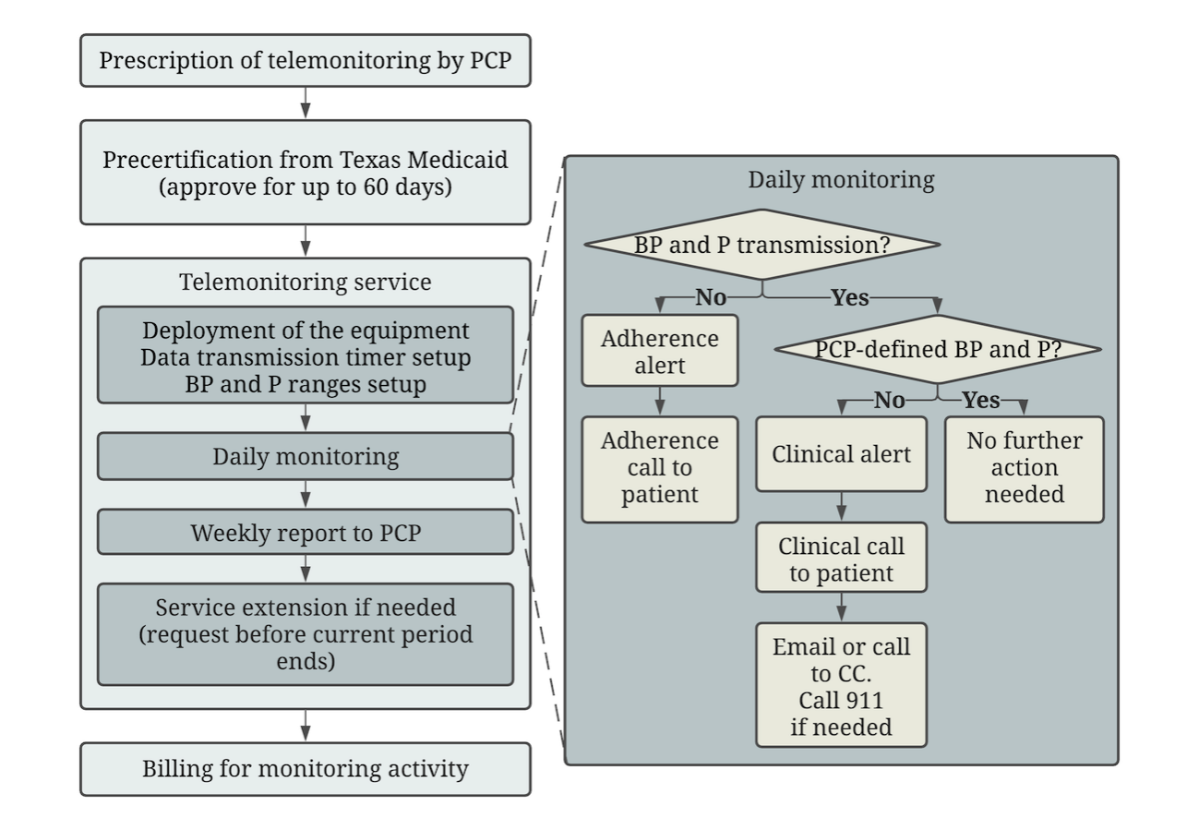
Workflow processes for telemonitoring service. BP: blood pressure; CC: clinical contact; P: pulse; PCP: primary care physician.

In this study, a telemonitoring company provided historical telemonitoring data (from January 2016 to December 2018) for Medicaid clients with hypertension in the state of Texas. The monitoring protocol (Figure 1) required patients to be referred by their physician. After Medicaid approval, a company technology deployer visited the patient’s home to set up the equipment and provide training. The equipment—Food and Drug Administration–approved devices—consisted of a monitoring device with Bluetooth technology and a signal transmission unit that transferred the monitoring results to the company’s cloud storage. No internet connection or smartphone was required. The training protocols and materials were developed based on American Medical Association guidelines [16]. The patients received education on how to use the equipment to take proper readings and were informed about the company’s protocols for responding to the patients’ technical or clinical needs. The patients were asked to select a daily time by which they would check and transmit their readings. If transmission did not occur by that time, an automated alert prompted a company staff member to make an adherence reminder call to the patient to troubleshoot any technical issues and to ask the patient to check and transmit the readings. Once the patient’s data were received, if the blood pressure reading or pulse rate fell outside the physician-defined acceptable ranges, an automated clinical alert was transmitted to a company nurse. The nurse placed a clinical phone call to the patient, categorized the extent of concern, and contacted the provider by email for the lowest level of concern and by both email and phone call for more severe concerns. The company provided weekly summary reports to each provider for the enrolled patients. Under Texas Medicaid rules, a request for reauthorization of the telemonitoring therapy was made every 60 days when the physician prescribed additional monitoring. Texas Medicaid paid as much as US $1074.60 for 60 days of monitoring [12,13].

### Data and Design

The telemonitoring company provided historical telemonitoring data for Texas Medicaid clients using their service. Only clients with 180 days or more of home telemonitoring were included in this study. The first 30 days were regarded as a startup period during which the patients learned to use the equipment to measure their vital signs. Data from the first 30 days were excluded from this study; thus, the study period was 150 days (months 1-5).

The number of transmissions before and after the adherence reminder calls was recorded each day, as was the number of adherence reminder calls made. We included all attempted adherence reminder calls, even those that the patients did not answer, because, in these cases, voice mail was left whenever possible. Daily systolic and diastolic blood pressure values were also collected to investigate improvements in blood pressure values during the study period. The mean systolic and diastolic blood pressure values at month 5 for each patient were calculated and compared with those at month 1. If the blood pressure values of the patient were missing for the entire month, that patient was excluded from this analysis.

The patients were separated into adherent and nonadherent cohorts; adherent patients were those who transmitted blood pressure and pulse values on at least 120 of the 150 days (at least 80% of the days, the same threshold used in the study by Milot et al [10]).

This study was approved by the institutional review board of Texas A&M University.

### Statistical Analysis

To determine whether the patient baseline characteristics differed by population subgroups, we used chi-square tests for categorical variables and two-tailed *t* tests for continuous variables. In addition, z tests for the equity of the two proportions were performed to examine whether the rates of transmission differed by population subgroups. Paired *t* tests were performed to analyze the changes in blood pressure values at month 5 by comparing them with those at month 1 for each subgroup. Independent *t* tests were used to compare the changes in blood pressure values between the population subgroups. Analyses were conducted using SAS version 9.4 (SAS Institute).

## Results

### Patient Characteristics

The data of 2093 clients enrolled in hypertension telemonitoring were provided. Of the 2093 patients, 1325 (63.31%) transmitted data at least once, and 823 (39.32%) transmitted data throughout a continuous 180-day period. Table 1 summarizes their characteristics. The mean age of the participants was 73.2 (SD 11.7) years, and 65.1% (536/823) were women. All patients included in this study were diagnosed with hypertension and were on pharmaceutical therapy.

**Table 1:** Demographics and nonalert ranges for overall, adherent, and nonadherent cohorts (N=823).

Characteristics		Patients		
		Overall (N=823)	Adherent (n=475)	Nonadherent (n=348)
Age (years), mean (SD)		73.2 (11.7)	73.8 (10.9)	72.3 (12.6)
Women, n (%)		536 (65.1)	301 (63.4)	235 (67.5)
**Area of residence^a^** **, n (%)**				
	Dallas	34 (4.1)	13 (2.7)	21 (6)
	Houston	26 (3.2)	11 (2.3)	15 (4.3)
	McAllen	648 (78.7)	368 (77.5)	280 (80.5)
	San Antonio	115 (14)	83 (17.5)	32 (9.2)
**Urban-rural classification^a^** **, n (%)**				
	Urban	660 (80.2)	360 (75.8)	300 (86.2)
	Suburban or rural	163 (19.8)	115 (24.2)	48 (13.8)
**Assigned nonalert range for systolic blood pressure, n (%)**				
	Default (90-160 mm Hg)	731 (88.8)	424 (89.3)	307 (88.2)
	Personalized	92 (11.2)	51 (10.7)	41 (11.8)
**Assigned nonalert range for diastolic blood pressure, n (%)**				
	Default (60-90 mmHg)	725 (88.1)	422 (88.8)	303 (87.1)
	Personalized	98 (11.9)	53 (11.2)	45 (12.9)
**Assigned nonalert range for pulse, n (%)**				
	Default (60-120 bpm)	724 (88)	421 (88.6)	303 (87.1)
	Personalized	99 (12)	54 (11.4)	45 (12.9)

^a^*P*<.001. Patient characteristics differ between adherent and nonadherent cohorts.

Most of the participants (648/823, 78.7%) were from McAllen in south Texas near the Mexican border, and most of them (660/823, 80.2%) resided in urban areas. Of the 823 participants, 731 (88.8%), 725 (88.1%), and 724 (88%) participants had acceptable systolic blood pressure, diastolic blood pressure, and pulse ranges of 90-160 mm Hg, 60-90 mm Hg, and 60-120 bpm, respectively, which were defined by their primary care physician. The remaining 92 (11.2%), 98 (11.9%), and 99 (12%) participants had customized acceptable values above or below these ranges (55-200 mm Hg, 50-120 mmHg, and 50-120 bpm for systolic blood pressure, diastolic blood pressure, and pulse ranges, respectively). Table 1 also provides descriptive characteristics of the adherent and nonadherent cohorts. The characteristics across the two cohorts were similar, although the adherent cohort had a higher proportion of suburban or rural patients, with more of them living in south Texas (*P*<.001).

### Adherence

**Figure 2:**
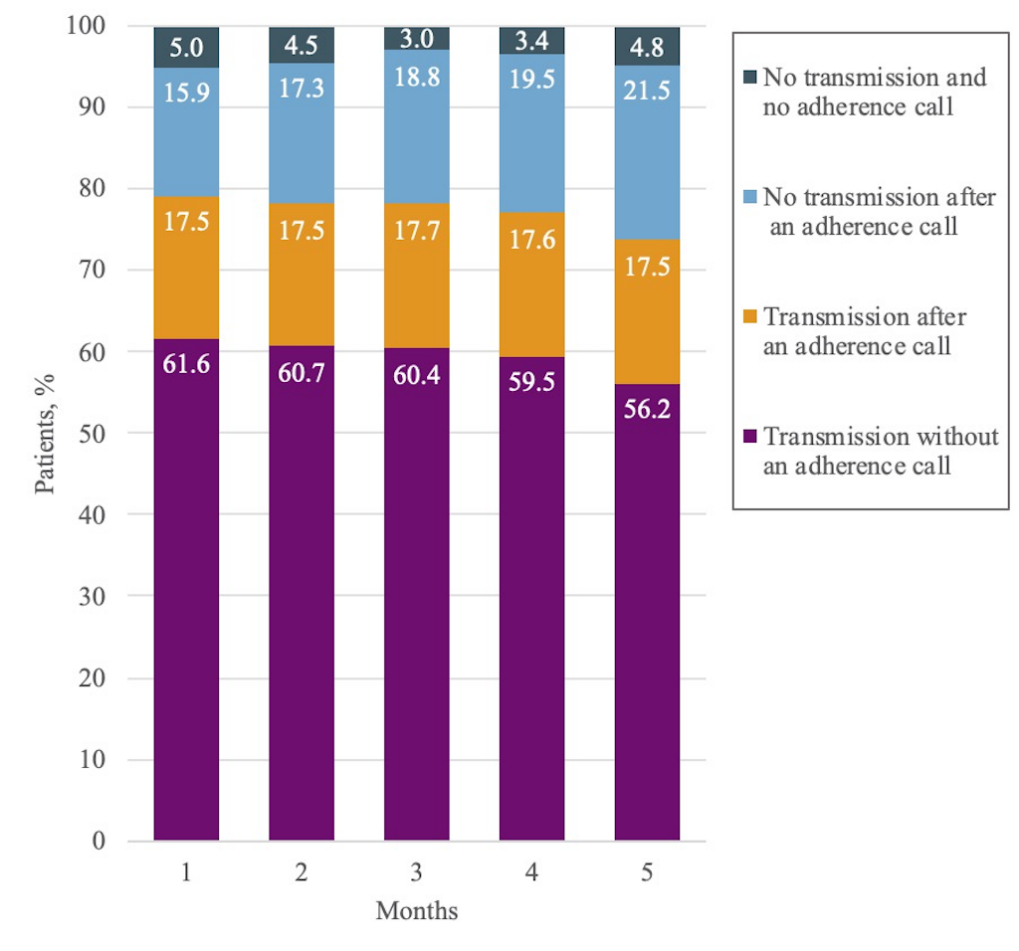
Monthly transmission rates for all patients over 150 days of telemonitoring. Mean transmission rate before (after) adherence call: 59.7% (77.2%).

Figure 2 shows the transmission rates (calculated using the following formula: transmission rate = 100 × total number of patients who transmitted readings / 823 patients) over the 5-month (150-day) period. The overall mean transmission rates across all 5 months were 59.7% before the adherence reminder call and 77.2% after the call (*P*<.001). The mean transmission rates for the first month were 61.6% and 79.1% before and after the call, respectively. These values declined until the fifth month when they reached 56.2% and 73.7% before and after the call, respectively. As indicated by the orange area in Figure 2, an average of 17.6% of the data transmissions were received after an adherence reminder call. However, the percentage of participants not transmitting after an adherence reminder call increased from 15.9% in the first month to 21.5% in the fifth month.

**Figure 3:**
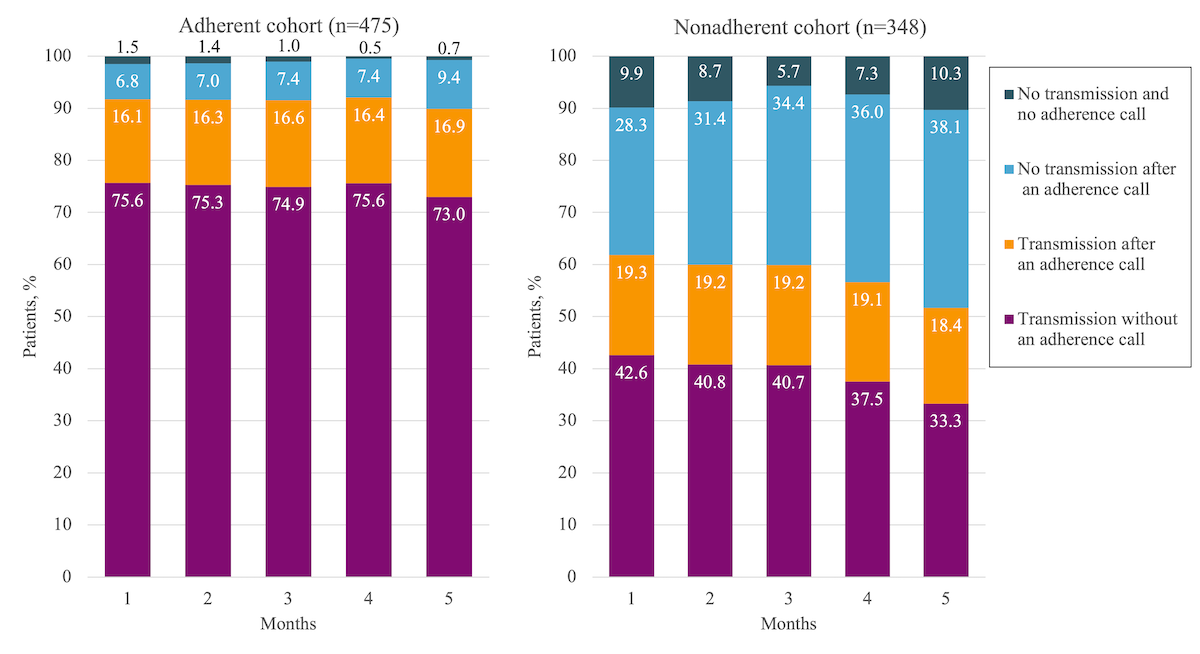
Monthly transmission rates for the adherent and nonadherent cohorts over 150 days of telemonitoring. Mean transmission rate before (after) adherence call: adherent cohort, 74.9% (91.3%); nonadherent cohort, 39% (58%).

These aggregate findings mask large differences between the adherent and nonadherent cohorts (Figure 3). The adherent cohort was much more likely to transmit data without an adherence reminder call, with an overall mean transmission rate of 74.9% compared with only 39% for the nonadherent cohort (*P*<.001). After the adherence reminder call, these values increased to 91.3% and 58% (*P*<.001), respectively.

The mean transmission rates for the first month were 75.6% before the adherence reminder call and 91.7% after the call for the adherent cohort and 42.6% and 61.9% before and after the call, respectively, for the nonadherent cohort. These values fluctuated and declined until the fifth month when they reached 73% and 89.9% before and after the call, respectively, for the adherent cohort and 33.3% and 51.7% before and after the call, respectively, for the nonadherent cohort. On average, an additional 16.5% and 19% transmissions were received after an adherence reminder call from the adherent and nonadherent cohorts, respectively (*P*<.001).

The percentage of participants not transmitting after an adherence reminder call was, on average, 7.6% for the adherent cohort and 33.6% for the nonadherent cohort. These values increased from 6.8% in the first month to 9.4% in the fifth month for the adherent cohort and from 28.3% in the first month to 38.1% in the fifth month for the nonadherent cohort (*P*<.001). We noted that, on average, 8.4% of the nonadherent participants who did not transmit data by the specified time failed to receive an adherence reminder call. This value increased to 10.3% in the fifth month of monitoring. In contrast, only 1.02% of the adherent cohort who did not transmit data failed to receive an adherence reminder call.

As might be expected, adherence was lowest on weekends (Tables 2 and 3), especially on Sundays, when the transmission rate (after the adherence reminder call) dropped to 88.4% and 46.3% for the adherent and nonadherent cohorts, respectively. The Sunday transmission rate was also observed to decrease over the 5-month period from 88.7% to 87.3% for the adherent cohort and from 49.6% to 41.8% for the nonadherent cohort.

**Table 2:** Weekday adherence by month for the adherent cohort (N=475).

		Adherence (%)						
		Sunday	Monday	Tuesday	Wednesday	Thursday	Friday	Saturday
**Month**								
	Month 1	88.7	92.9	93.3	93.4	92.5	91.8	90
	Month 2	87.8	92.5	92.8	92.1	92.6	92.7	90.8
	Month 3	88.4	91.9	93.1	92.9	92.8	92.4	89.1
	Month 4	89.6	92.9	91.8	93.4	93.1	93.1	90.5
	Month 5	87.3	91.5	91	90.4	90.7	90.1	88.4
Value, mean (SD)		88.4 (0.8)	92.3 (0.6)	92.4 (0.9)	92.4 (1.1)	92.3 (0.8)	92 (1)	89.8 (0.9)

**Table 3:** Weekday adherence by month for the nonadherent cohort (N=348).

		Adherence (%)						
		Sunday	Monday	Tuesday	Wednesday	Thursday	Friday	Saturday
**Month**								
	Month 1	49.6	66.9	67.1	68.6	65.6	63	52.8
	Month 2	47.9	64.9	64.2	64.9	64.9	61.8	51.7
	Month 3	46.7	64.4	64.1	65.2	63.8	62.1	52.1
	Month 4	45.7	60.2	61	61.6	59.9	57.7	50.1
	Month 5	41.8	55	55.5	56	55.6	54.2	44
Value, mean (SD)		46.3 (2.6)	62.3 (4.2)	62.4 (3.9)	63.3 (4.2)	62 (3.7)	59.7 (3.3)	50.1 (3.2)

Along with adherence to the daily protocol, the data also indicated whether the transmissions received were in or out of the physician-specified range. The average percentage of transmissions in range (calculated using the following formula: average percentage of transmissions in range = 100 × [number of transmissions in range / total number of transmissions]) was found to be 60.9% (SD 26%) for the adherent cohort and 53.9% (SD 24.9%) for the nonadherent cohort. The percentage in range increased for both cohorts over the 5-month period, indicating that telemonitoring was effective, from 59.2% in month 1 to 62.3% in month 5 for the adherent cohort and 49.8% in month 1 to 56.7% in month 5 for the nonadherent cohort.

Finally, the data indicated that the transmission results for 2 consecutive days were related. Note that for any given day, there were three possible outcomes: the patient did not transmit, the patient transmitted an out-of-range reading (blood pressure values, pulse rate, or both) or the patient transmitted an in-range reading. We refer to these as *transmission events*. Frequency analysis indicated an association between the transmission events observed on consecutive days. This is explored in the following sections. The percentages are listed in Table 4.

**Table 4:** Next day transition (N=823).

From	To (%)						
	Adherent cohort (n=475)				Nonadherent cohort (n=348)		
	NT^a^	ORT^b^	IRT^c^	NT		ORT	IRT
NT	32.9	29.3	37.8	61.7		18.5	19.8
ORT	5.6	41.6	52.7	28.9		34.8	36.3
IRT	6.3	32	61.7	29.6		28	42.5

^a^NT: no transmission.

^b^ORT: out-of-range transmission.

^c^IRT: in-range transmission.

### Trends in Transmission Events Between 2 Consecutive Days

Adherence on the day after a missed transmission was far below the overall average for both adherent (67.1% vs an average of 91.3%) and nonadherent (38.3% vs an average of 58%) cohorts. Furthermore, the transmissions that were received the day after a missed transmission were less likely to be in range than the average for both cohorts. For the adherent cohort, 37.8% of the missed transmissions were followed by in-range transmissions, indicating that 56.3% (100 × [37.8 / 67.1]) of the transmissions received the day after a missed transmission were in range, whereas for the nonadherent cohort, 19.8% of the missed transmissions were followed by in-range transmissions, indicating that 51.6% (100 × [19.8 / 38.3]) of the transmissions received the day after a missed transmission were in range.

Adherence and in-range transmission after out-of-range transmission also showed similar patterns across the 2 cohorts. For the adherent cohort, out-of-range transmissions were followed by 41.6% of out-of-range transmissions, 52.7% of in-range transmissions, and only 5.6% of no transmissions the next day. For the nonadherent cohort, out-of-range transmissions were followed by 34.8% of out-of-range transmissions, 36.3% of in-range transmissions, and 28.9% of no transmissions the next day. Thus, adherence after an out-of-range day was greater than the overall average (94.6% vs an average of 91.3% for the adherent cohort and 71.1% vs an average of 58% for the nonadherent cohort). Furthermore, the transmissions that were received after an out-of-range transmission were less likely to be in range than the overall average (55.7%—100 × [52.7 / 94.6]—vs an average of 60.9% for the adherent cohort and 51%—100 × [36.3 / 71.1]—vs an average of 53.8% for the nonadherent cohort). It is worth noting that when an adherent patient transmitted an out-of-range reading, the next in-range transmission occurred within 2-3 days on average, that is, it took 2-3 days to resolve whatever problem was causing the out-of-range reading and for the patient to regain blood pressure and pulse rate control. However, when a nonadherent patient transmitted an out-of-range reading, the next in-range transmission did not occur for 5-6 days on average, indicating that nonadherent patients were likely to experience elevated levels of blood pressure or pulse rate over a longer period.

Finally, adherence and in-range transmission after an in-range transmission also had similar patterns across the 2 cohorts, with better adherence and more in-range transmissions on the following day. For the adherent cohort, in-range transmissions were followed by 61.7% of in-range transmissions, 32% of out-of-range transmissions, and only 6.3% of no transmissions the next day. For the nonadherent cohort, in-range transmissions were followed by 42.5% of in-range transmissions, 28% of out-of-range transmissions, and 29.6% of no transmissions the next day. Thus, adherence after an in-range day was greater than the overall average (93.7% vs an average of 91.3% for the adherent cohort and 70.5% vs an average of 58% for the nonadherent cohort). Furthermore, the transmissions that were received after an in-range transmission were more likely to be in-range again the next day than the overall average (65.8%—100 × [61.7 / 93.7]—vs an average of 60.9% for the adherent cohort and 60.3%—100 × [42.5 / 70.5]—vs an average of 53.8% for the nonadherent cohort).

### Relationship Between Daily Adherence and Blood Pressure Control

Overall, we found that the systolic blood pressure values of the adherent cohort improved by an average of 2.2 mm Hg (*P*<.001) over 5 months, whereas those of the nonadherent cohort improved by an average of 1.6 mm Hg (*P*=.02; Table 5). This improvement in the adherent cohort was significantly higher than that in the nonadherent cohort (*P*=.049).

**Table 5:** Systolic blood pressure changes between month 1 and month 5 (n=781).

			Adherent cohort (n=475; mm Hg)		Nonadherent cohort (n=306^a^; mm Hg)		*P* value^b^
**Month 1**							
	Value, mean (SD)	133.7 (12.5)		137.9 (15.0)		N/A^c^	
**Month 5**							
	Value, mean (SD)	131.4 (12.2)		136.3 (14.4)		N/A	
**Comparison between month 1 and month 5**							
	Value, mean (SD)	2.2 (9.5)		1.6 (12.0)		.049	
	*P* value^d^	<.001		.02		N/A	

^a^A total of 42 patients were excluded because of missing data.

^b^A two-tailed independent *t* test was performed to compare the systolic blood pressure changes between the adherent and nonadherent cohorts.

^c^N/A: not applicable.

^d^A two-tailed paired *t* test was performed to analyze the differences in systolic blood pressure values between month 1 and month 5 for each cohort.

Furthermore, of the 21 patients with an average systolic and diastolic blood pressure reading of more than 140 and 90 mm Hg for the first month, we found that the systolic blood pressure of the adherent patients (7/21, 33%) improved by an average of 14.8 mm Hg (*P*=.02) over 5 months, whereas that of the nonadherent patients (14/21, 67%) improved by an average of 10.6 mm Hg over 5 months, which was not significantly different (*P*=.11). The diastolic blood pressure of the adherent patients improved by an average of 0.7 mm Hg (*P*=.004) over 5 months, whereas the improvement over 5 months was not significant for nonadherent patients (0.4 mm Hg; *P*=.39; Table 6).

**Table 6:** Diastolic blood pressure changes between month 1 and month 5 (n=781).

Month			Adherent cohort (n=475; mm Hg)		Nonadherent cohort (n=306^a^; mm Hg)		*P* value^b^
**Month 1**							
	Value, mean (SD)	71.5 (7.9)		74.0 (10.0)		N/A^c^	
**Month 5**							
	Value, mean (SD)	70.7 (7.9)		73.6 (9.8)		N/A	
**Comparison between month 1 and month 5**							
	Value, mean (SD)	0.7 (5.6)		0.4 (7.9)		.09	
	*P* value^d^	.004		.39		N/A	

^a^A total of 42 patients were excluded because of missing data.

^b^A two-tailed independent *t* test was performed to compare the diastolic blood pressure changes between the adherent and nonadherent cohorts.

^c^N/A: not applicable.

^d^A two-tailed paired *t* test was performed to analyze the differences in diastolic blood pressure between month 1 and month 5 for each cohort.

Of the 21 patients with an average systolic and diastolic blood pressure reading of more than 140 and 90 mm Hg for the first month, we found that the diastolic blood pressure of adherent patients (7/21, 33%) improved by an average of 11.0 mm Hg (*P*=.02) over 5 months, whereas that of the nonadherent patients (14/21, 67%) improved over 5 months by an average of 9.7 mm Hg (*P*=.03).

## Discussion

### Principal Findings

This study suggests that telemonitoring for hypertension can achieve more than 70% adherence among Medicaid clients. Thus, most patients should be able to check and transmit their blood pressure values and pulse rate after the initial training. Furthermore, much higher levels of adherence (up to 90%) are possible for most patients (475/823, 57.7% of the patients in this study had 80% or more days of transmission) when telemonitoring is accompanied by adherence reminder calls. For these patients, adherence levels seemed to decline slightly over the 5-month period.

Furthermore, many Medicaid patients are likely to have trouble with daily adherence (348/823, 42.3% of the patients in this study). For these patients, adherence reminder calls can be helpful, but many daily transmissions will still be missed (approximately 13 days per patient per month in this study). Such patients can likely be identified within the first month of monitoring (not including the startup period), when their adherence rates without the adherence reminder call fall well below 50% (42.6% in this study). Indeed, 75% (260/348) of the patients in the nonadherent cohort in this study were not adherent in the first month of monitoring. For these patients, adherence rates can be expected to degrade significantly over time (by approximately 16% over 5 months in this study). Of the 823 patients, the 475 (57.7%) adherent patients and the 348 (42.3%) nonadherent patients together generated the need for approximately 350 adherence reminder calls per day, a significant workload. Regardless of the case, patients with adherence problems clearly need more than an adherence reminder call. Indeed, interventions that delve into health behaviors will likely be necessary (but perhaps not sufficient) to bring adherence levels up to 80% and beyond. The data suggest that such interventions should be targeted to weekends and to days after missed transmissions when the likelihood of poor adherence is higher.

Just as additional support for better adherence to daily monitoring is necessary, follow-up on an abnormal clinical condition is also important. A potential benefit of daily monitoring is that health care providers may recognize and address emerging problems before they become urgent. When readings are not transmitted, this opportunity may be lost. If we assume that the percentage of out-of-range transmissions can be applied to the days when data were not transmitted, we can estimate the number of missed transmissions that would have been out of range. Over 150 days of monitoring, this estimate turned out to be 5.1 days ([1 – 0.913] × [0.391] × 150) per patient for the adherent cohort and 29.0 days ([1 – 0.580] × [0.461] × 150) per patient for the nonadherent cohort. This represents a total of 12,528 days (29.04 × 348 + [5.10 × 475]) of unmet needs for 823 patients over 150 days of monitoring (approximately 15 days per patient). In other words, 10.2% (12,528 / [150 × 823]) of the required follow-up was missed because of lack of adherence (for the nonadherent cohort, this was approximately 20%).

On a positive note, it is encouraging that 58.9% (280/475) of the adherent patients and 54.9% (168/306) of the nonadherent patients experienced an improvement in systolic blood pressure values, and 52.2% (248/475) of the adherent patients and 51.6% (158/306) of the nonadherent patients experienced an improvement in diastolic blood pressure values. The mean systolic blood pressure values of both cohorts improved significantly during the study period, and these improvements were significantly higher in the adherent cohort (*P*=.049). The mean diastolic blood pressure value of the adherent cohort declined significantly during the study period, but the decline was not significant for the nonadherent patients. These results are consistent with those of clinical trials in the literature. In 18 clinical trials of home telemonitoring, the average improvement in systolic and diastolic blood pressure values was 12.1 and 6.3 mm Hg within 6 months [17-34]. Of these 18 clinical trials, eight were restricted to patients with systolic and diastolic blood pressure values of more than 140 and 90 mm Hg at baseline, and the other ten trials were restricted to those with blood pressure readings above or below these values. In our study with patients with systolic and diastolic blood pressure values of more than 140 and 90 mm Hg in the first month, the systolic and diastolic blood pressure values of the adherent patients improved by an average of 14.8 and 11.0 mm Hg, which is higher than the average improvement observed in the 18 clinical trials. However, for nonadherent patients with systolic and diastolic blood pressure values of more than 140 and 90 mm Hg in the first month, only the diastolic blood pressure value significantly improved by an average of 9.7 mm Hg.

Finally, it is important to appreciate that achieving improved adherence requires considerable effort. Patients must be trained in the correct procedures to monitor their blood pressure and pulse; staff members must monitor daily transmissions and contact patients to encourage participation and to resolve technical issues; and, as noted, additional interventions will be needed for many patients. Texas Medicaid payment levels may have been adequate for this level of intervention, but it is not clear whether Medicare or private insurers will reimburse this level of effort in the future. Clearly, the case for reimbursement would be compelling if hypertension telemonitoring could be shown to help avoid even a small number of hospitalizations for stroke and heart disease, which can be extremely expensive.

This study included some limitations. It only examined Texas Medicaid clients. It is not clear whether these findings are generalizable to Medicare, privately insured, or uninsured patients with hypertension. It is also unclear whether these findings are generalizable to people with other chronic conditions who would benefit from ongoing monitoring. This study was limited to patients who were referred to the monitoring program. The analysis would be strengthened if there were a control group to more rigorously examine adherence and the impact of the intervention. Finally, the monitoring protocol required the data to be transmitted on a daily basis, which was more frequent than the general home blood pressure monitoring guidelines [5]. Excessive and frequent transmission requirements may negatively affect adherence and persistence. In contrast, daily monitoring could help with medication adherence and help avert emergency situations and hospitalizations.

### Conclusions

Adherence reminder calls helped most patients with hypertension to achieve higher levels of adherence to blood pressure and pulse monitoring. Telemonitoring improved blood pressure control, similar to the improvement observed in clinical trials. Furthermore, more adherent patients achieved higher levels of blood pressure control. However, the study suggests that additional adherence interventions and support are needed for many patients to achieve high levels of adherence.
