# Correction: Adherence to Telemonitoring Therapy for Medicaid Patients With Hypertension: Case Study

**DOI:** 10.2196/39666

**Published:** 2022-06-17

**Authors:** Sulki Park, Hye-Chung Kum, Michael A Morrisey, Qi Zheng, Mark A Lawley

**Affiliations:** 1 Population Informatics Lab Texas A&M University College Station, TX United States; 2 Department of Industrial and Systems Engineering Texas A&M University College Station, TX United States; 3 Department of Health Policy and Management Texas A&M University College Station, TX United States; 4 Department of Epidemiology and Biostatistics Texas A&M University College Station, TX United States

In “Adherence to Telemonitoring Therapy for Medicaid Patients With Hypertension: Case Study” (J Med Internet Res 2021;23(9):e29018), the authors noticed one error.

In the originally published article, the Acknowledgments section inadvertantly missed a statement acknowledging the telemonitoring company that provided the real-world data for the study. In the corrected version of the article, the following sentence has been added to the Acknowledgments section:

Real-world telemonitoring data was provided by Coordination Centric.

The correction will appear in the online version of the paper on the JMIR Publications website on June 17, 2022, together with the publication of this correction notice. Because this was made after submission to PubMed, PubMed Central, and other full-text repositories, the corrected article has also been resubmitted to those repositories.

